# Performance of Bearing Ball Defect Classification Based on the Fusion of Selected Statistical Features

**DOI:** 10.3390/e24091251

**Published:** 2022-09-05

**Authors:** Zahra Mezni, Claude Delpha, Demba Diallo, Ahmed Braham

**Affiliations:** 1 Ecole Nationale Supérieure d’Ingénieurs de Tunis (ENSIT), University of Tunis, Tunis 1007, Tunisia; 2 The Matériaux, Mesures et Applications (MMA) Laboratory, University of Carthage, Carthage 1054, Tunisia; 3 Laboratoire des Signaux et Systèmes, CNRS, CentraleSupelec, Université Paris Saclay, 91192 Gif sur Yvette, France; 4 Group of Electrical Engineering of Paris, CNRS, CentraleSupelec, Université Paris Saclay, 91192 Gif sur Yvette, France

**Keywords:** bearing ball fault, classification, feature selection and extraction, empirical mode decomposition, statistical analysis, Kullback–Leibler divergence, machine learning

## Abstract

Among the existing bearing faults, ball ones are known to be the most difficult to detect and classify. In this work, we propose a diagnosis methodology for these incipient faults’ classification using time series of vibration signals and their decomposition. Firstly, the vibration signals were decomposed using empirical mode decomposition (EMD). Time series of intrinsic mode functions (IMFs) were then obtained. Through analysing the energy content and the components’ sensitivity to the operating point variation, only the most relevant IMFs were retained. Secondly, a statistical analysis based on statistical moments and the Kullback–Leibler divergence (KLD) was computed allowing the extraction of the most relevant and sensitive features for the fault information. Thirdly, these features were used as inputs for the statistical clustering techniques to perform the classification. In the framework of this paper, the efficiency of several family of techniques were investigated and compared including linear, kernel-based nonlinear, systematic deterministic tree-based, and probabilistic techniques. The methodology’s performance was evaluated through the training accuracy rate (TrA), testing accuracy rate (TsA), training time (Trt) and testing time (Tst). The diagnosis methodology has been applied to the Case Western Reserve University (CWRU) dataset. Using our proposed method, the initial EMD decomposition into eighteen IMFs was reduced to four and the most relevant features identified via the IMFs’ variance and the KLD were extracted. Classification results showed that the linear classifiers were inefficient, and that kernel or data-mining classifiers achieved 100% classification rates through the feature fusion. For comparison purposes, our proposed method demonstrated a certain superiority over the multiscale permutation entropy. Finally, the results also showed that the training and testing times for all the classifiers were lower than 2 s, and 0.2 s, respectively, and thus compatible with real-time applications.

## 1. Introduction

Electric actuators are increasingly present in several application areas such as transport, health or renewable energy. At the same time, the requirements for operational safety and energy efficiency are becoming increasingly stringent. In response, condition-based maintenance was introduced. It requires regular monitoring of all system components, including electrical machines. Electrical machines operating increasingly under severe conditions or at the limits of their capacity are subject to faults that can lead to failures [1,2,3]. Investigations in several industrial fields have revealed that rolling bearing elements (RBEs) are the main sources of failures for almost 40% to 90% of low- to high-power machines [4,5]. Several studies [6,7] have concluded that bearing ball fault is the most difficult to diagnose (detection and classification) because its effects are strongly attenuated by the mechanical structure.

Bearing balls can be affected by corrosion, spalling, high temperatures or premature wear due to overloading. In the long term, these degradations lead to holes in the balls.

It is of a paramount importance to investigate these types of faults considered as incipient ones and that can be found in such electrical machines (synchronous or asynchronous machines). In the considered testbed, three bearings were degraded. For each of them, a hole with a different diameter was drilled in a ball to evaluate the proposed diagnostic method.

It is usual to consider that fault detection and diagnosis methodologies can be summarised in four major steps: (1) modelling, (2) data preprocessing, (3) feature extraction and selection and (4) feature analysis for decision-making. The modelling is derived from physics-based models or historic data. Because accurate analytical models are not readily available, a data-driven approach has become more and more attractive thanks to the increasingly digitalised systems. Vibration signals are still considered to contain the most relevant information about the health of the bearings [8,9,10,11]. In fact, the main advantages of vibration-based diagnosis is its ability to detect different types of defects, either distributed or localised [9].

Before the extraction and analysis of the most representative fault features, raw time-series vibration signals need to be preprocessed [8,12,13]. In this step, several operations can be done depending on the fault detection requirements, the quantity of data and the signal properties. One of the main operation consists in selecting the most appropriate space of representation: time domain, frequency domain, or time–frequency domain [14]. The varying operating conditions lead to transient and nonstationary signals [15,16]. Therefore, time-domain features and classical frequency-domain techniques such as FFT fail to diagnose bearing faults. Over the years, several time–frequency (and time–scale) techniques such as the short-time Fourier transform (STFT) [17,18], wavelet transform (WT) and its derivatives namely the Wavelet packet transform (WPT), discrete wavelet transform (DWT) [19,20], and Hilbert–Huang transform (HHT) [21,22] have been used to address this issue. Nonetheless, the STFT suffers from a fixed window length, while the performance of the WT strongly depends on the selection of the mother wavelet and the decomposition level. Moreover, both methods are limited by the two contradictory targets that cannot be reached simultaneously: a high time and frequency resolution [23].

In the past few decades, other decomposition techniques have been applied to vibration time series [24,25], among them, the empirical mode decomposition (EMD) [26,27], ensemble empirical mode decomposition (EEEMD) [28,29,30] or variational mode decomposition (VMD) [31,32,33]. Despite its shortcomings such as mode mixing and end effects [27,34,35], the EMD is still very popular. In fact, EMD decomposes a time-series signal into a finite number of signals denoted as intrinsic mode functions (IMF), and a residue. Each IMF represents the original signal in a frequency band ranging from high to low frequencies. Compared to the original signal, the IMFs can better describe the intrinsic properties of the raw data [36]. However, the fault information is not evenly distributed among the IMFs.

Therefore, it is of utmost importance to select the most relevant IMFs and consider the best features in order to obtain the highest detection and classification rates. Moreover, the retained IMFs should also be robust to nuisances (environmental noise) and variable operating conditions. In the literature, most of the studies focus on the classification between inner race, outer race, cage and ball faults [11,12]. However, the classification of ball faults based on their severities is more tedious and still an open research topic. This paper addresses this problem and highlights the performance according to the chosen family of classification techniques. Indeed, if we consider that when using statistical clustering techniques, several solutions can be considered according to the nature of the data, their linearity, their separability, and so forth, then the techniques can be separated in different families. With no loss of generality, we can mention: linear projection techniques such as the principal component analysis [37] (PCA); kernel-based nonlinear techniques such as the kernel principal component analysis (KPCA), support vector machines (SVMs) [38]; kernel estimation techniques such as the K-nearest neighbours (KNN) algorithm [39]; tree-based techniques such as decision trees (DTs) [40]; or probabilistic techniques such as the naive Bayes (NB) classifier [41]. For the ball bearing classification problem, we propose to evaluate and compare the efficiency of these techniques.

To the best of our knowledge, a piece of equipment using vibration signal analysis for industrial applications provides general information (fault isolation and a rough estimate of the severity of the defect) on mechanical defects: loosening, rotor unbalance, bearings or misalignment. The most usual professional bearing condition monitoring systems are based on the characteristics of specific harmonics (RMS, frequency) extracted from time series of vibration signals. However, the results could be biased due to ageing, variable load conditions and environmental nuisances. The proposed method detailed in this work complements the available instruments since it allows a more accurate analysis of bearing defects, in this case, balls. The proposed methodology allows a more accurate diagnosis up to the classification with information extracted from vibration data, which is so far the most popular in the industry. Thanks to the preprocessing, the proposed method extracts fault characteristics (statistical and distance-based features) that are the most sensitive to the defect and robust to the nuisance and load variations. Processing times are also compatible with real-time application. The proposed method is also in line with the growing use of machine learning techniques.

The organisation of the paper is as follows: Section 2 highlights the paper contribution, Section 3 describes the proposed ball fault diagnosis methodology. The EMD and the basics of each data representation technique are shortly reviewed, and the extracted features are introduced. Section 4 is devoted to the experimental evaluation of the classifiers using the CWRU database. The conclusion and future research are presented in Section 5.

## 2. Paper Contribution

In the following, we propose a four-step methodology to perform ball fault classification with the combination of properly selected statistical features. The vibration signals used to illustrate the rational of the approach were obtained from the Case Western Reserve University database [42]. In the preprocessing step, the EMD was adopted to decompose the original signal into IMFs. Then, the most relevant ones were retained from the analysis of their signal-to-noise ratio and their invariance to the operating conditions. The fault features selected were derived from their first four statistical moments (i.e., mean, variance, skewness, kurtosis) and their probability density functions (PDF) using the Kullback–Leibler divergence [43,44,45]. The selection process identified the most relevant features as the IMFs’ variance and the KLD. For the fault classification, the selected features were merged then analysed with several techniques including the traditional PCA, KPCA, SVM, and data exploration solutions with deterministic (KNN and DT) and probabilistic (NB) approaches. The classification performance of all these techniques was evaluated and compared in terms of training accuracy rate (TrA), testing accuracy rate (TsA), training time (Trt) and testing time (Tst). The most suitable classification technique was then highlighted and compared to the literature’s main results for this particular incipient fault diagnosis.

## 3. The Fault Diagnosis Methodology

**Figure 1:**
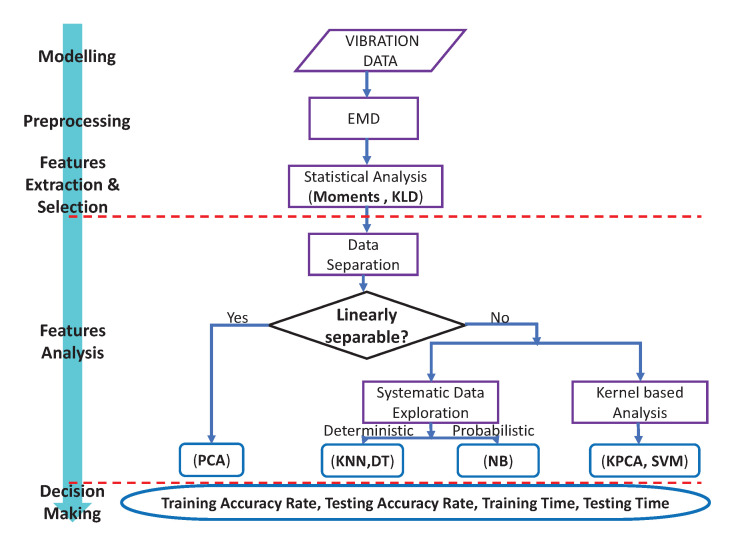
Ball fault diagnosis methodology.

Figure 1 gives an overview of the methodology with the four steps.

### 3.1. Preprocessing and Feature Extraction and Selection

The first step, modelling, assumed the use of vibration raw data. With no loss of generality, the raw data used here were vibration time- dependent information recorded with a sampling frequency of 12 kHz using accelerometers located at 12 o’clock on the housing of the drive end and the fan end. This is detailed in Section 4.1, which describes the experimental data. Note that the data corresponded to the healthy and three faulty cases (0.007, 0.014, 0.021 inch) with four different load conditions: no load (L0), half-load (L1), full load (L2), overload (L3). These load conditions allowed us to clearly observe the influence of the machine’s torque.

For the second step, preprocessing, the raw vibration signals were decomposed into intrinsic mode functions (IMFs) using the EMD. In order to retain the most relevant IMFs [34] from the 18 IMFs, the relative deviation percentage (RDP) of the IMFs’ signal-to-noise ratio (SNR) was computed [46,47]. Table 1 presents the results.

IMFs with an SNR RDP greater than 40 dB were considered irrelevant as they were highly sensitive to the noise. As a consequence, only the first eight IMFs were considered for the bearing ball fault diagnosis. It was also proved in [47] that the first IMF ($IMF_{1}$) was very sensitive to the load variation. Finally, only $IMF_{2}$ to $IMF_{8}$ were retained for the feature extraction.

**Figure 2:**
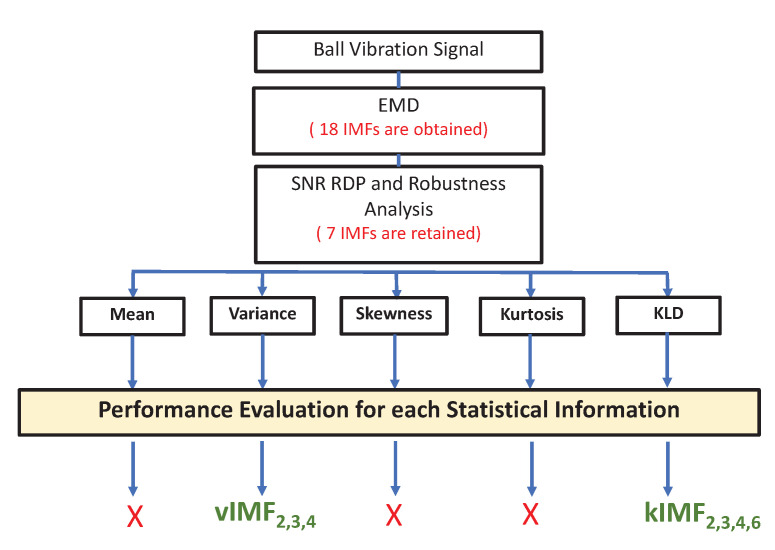
IMF selection and feature extraction procedure.

As depicted in Figure 2, in the feature extraction process, we analysed the first four statistical moments and the Kullback–Leibler divergence (KLD) of these seven IMFs for different operating conditions. It was found that [46,47]:

- The mean and the skewness had very poor detection performance
- The kurtosis had a very low sensitivity to the ball fault level.

Using the receiver operating characteristic (ROC) curve [48], the area under curve (AUC) was computed as an efficiency criterion to proceed with the feature selection. In Table 2, the AUC values are summarized for the four statistical moments, the KLD and each retained IMF. It can be noticed that the most relevant features are selected whatever the operating condition $L_{0}$, $L_{1}$, $L_{2}$ and $L_{3}$.

It can be noticed that the KLD and variance for most of the IMFs are the main features to be retained whatever the operating condition. Furthermore, if the noise variation is considered, then the sensitivity of these features are kept for a reduced number of IMFs [46,47].

Finally, the most relevant features retained for the bearing ball extraction were as follows:

- **Variance**: $IMF_{2}$, $IMF_{3}$ and $IMF_{4}$; denoted as $vIMF_{j}$
- **KLD**: $IMF_{2}$, $IMF_{3}$, $IMF_{4}$ and $IMF_{6}$; denoted as $kIMF_{j}$.

Note that the subscript *j* indicates the rank of the considered IMF.

### 3.2. Feature Analysis

As shown in the flowchart from Figure 1, the choice of the appropriate classification technique depends on whether the data are linearly separable or not. In the following, a brief review of the classification methods proposed to be used in this work is presented.

#### 3.2.1. Principal Component Analysis (PCA)

The PCA is a linear unsupervised technique. It projects the original data into a lower dimension subspace while minimising the reconstruction error [37]. This method has in fact received considerable attention in the fault detection and diagnosis framework over the last three decades since no prior complex physical knowledge on the process is needed. The only information required is a history of data representing several operating conditions.

However, most of the processes exhibit nonlinear behaviour and the measurements are affected with noise whose distribution may be unknown. Therefore, linear methods such as PCA may have poor performance. In the following, improved techniques are investigated such as:

- Kernel-based techniques: kernel principal component analysis (KPCA) and support vector machine (SVM);
- Deterministic systematic exploration techniques: K-nearest neighbours (KNN) and decision tree (DT);
- Probabilistic systematic exploration techniques: naive Bayes classifiers (NB).

#### 3.2.2. Kernel Principal Component Analysis (KPCA)

KPCA was firstly proposed by Scholkop et al. [49] and was provided as an alternative to PCA, which allowed the nonlinear feature extraction from a dataset by using a specific kernel. As reported in the literature, this technique has been successfully used in fault detection and diagnosis (FDD) of several systems [50,51,52,53]. The main idea of this technique relies on mapping the data into a feature space through a nonlinear function (denoted as the kernel) so that PCA can be performed in that feature space. The kernel function is the core of the KPCA algorithm. It is a positive semidefinite function that introduces nonlinearity into the process. The most classic kernels considering two sample vectors *x* and *y* are:

- The polynomial kernel defined as ( $p\in R^{+}$ is the kernel’s order):
  (1) $$K\left(x,y\right)={\left(\langle x,y\rangle +1\right)}^{p}$$
- The Gaussian kernel defined as ( $\gamma \in R^{+}$ is the standard deviation of the kernel):
  (2) $$K\left(x,y\right)=exp\left(-\frac{{∥x-y∥}^{2}}{2\gamma^{2}}\right)$$

The performance of KPCA is strongly related to the selected kernel and to the tuning of its hyperparameters, which depends on the data distribution [38,54]. In the following, for each kernel, the performance is analysed with regard to the setting of each hyperparameter. Despite its advantages, KPCA is time- and memory-consuming as the size of the database increases with a third-order complexity $O(N^{3})$, where *N* is the data set dimension and required storage capacity for an (N×N) kernel matrix.

#### 3.2.3. Support Vector Machine (SVM)

Used for classification, regression and outlier detection, an SVM is considered as one of the most attractive supervised machine learning algorithms and was developed by Vapnick in 1995 [55]. Its basic idea consists of finding the highest dimension feature space in which the projected data are linearly separable with the highest margin between the different classes. This projection also requires the selection and tuning of kernel functions. Therefore, choosing the kernel, then adjusting its hyperparameters, is also a critical task as it highly impacts the classification’s performance. As for KPCA, famous and widely used kernels are polynomial ones (i.e., Equation (1)) and Gaussian ones (i.e., Equation (2)). Their tuning are both considered for this study.

#### 3.2.4. K-Nearest Neighbours (KNN)

KNN is a nonparametric, supervised, and easy to implement classification technique. This classifier is exclusively based on the selection of the classification metric, among which we can cite the Euclidean distance (Euc), the city block distance (CB), the Minkowsky distance (Mink) and the Chebyshev distance (Chb). The technique is based on distance estimation and the tuning of a predefined number of nearest neighbours denoted K [56]. In [57], the determination of K as well as the pertinent metrics were analysed. In the following, K was selected in the range of [1:10] and Euclidean and city block distances were evaluated. They are recalled in the following.

If we consider two vectors A and B defined, respectively, with samples $x_{i}$ and $y_{i}$ such as $A=(x_{1},x_{2},\ldots ,x_{m})$ and $B=(y_{1},y_{2},\ldots ,y_{m})$, the distance metrics are:

- *Euclidean distance* (Euc). It is defined by:
  (3) $$Euc\left(A,B\right)=\sqrt{\sum_{i=1}^{m}{\left(x_{i}-y_{i}\right)}^{2}}$$
- *City block distance* (CB) given as:
  (4) $$CB\left(A,B\right)=\sum_{i=1}^{m}|x_{i}-y_{i}|$$

#### 3.2.5. Decision Tree (DT)

A DT is considered as one of the most popular supervised machine learning techniques for solving data classification or regression problems. With its graphical tree-based geometry, the decisions are positioned at the ends of the branches also called leaves [58,59,60]. As in the KNN algorithm, a DT is also based on two main parameters: the maximum split number criterion (MSpN), and the split criterion (Spc). In the following, MSpN was set to 50 to avoid overfitting. The maximum deviance reduction (MDR), twoing rule (TR) and Gini’s split criteria were adopted in our approach to tune the Spc. More details can be found in [57].

#### 3.2.6. Naive Bayes (NB)

Naive Bayes is one of the powerful probability-based supervised machine learning classification method. It has been used in several applications due to its simple implementation, ease of physical interpretation, fast computation speed and excellent classification performance [61,62,63]. However, the assumption of conditional independence of variables may limit its performance when dealing with real vibration data.

## 4. Results and Discussions

In this section, first, we introduce the experimental testbed and the data. Then, we present the classification results for each of the method described in the previous section.

### 4.1. Experimental Data

**Figure 3:**
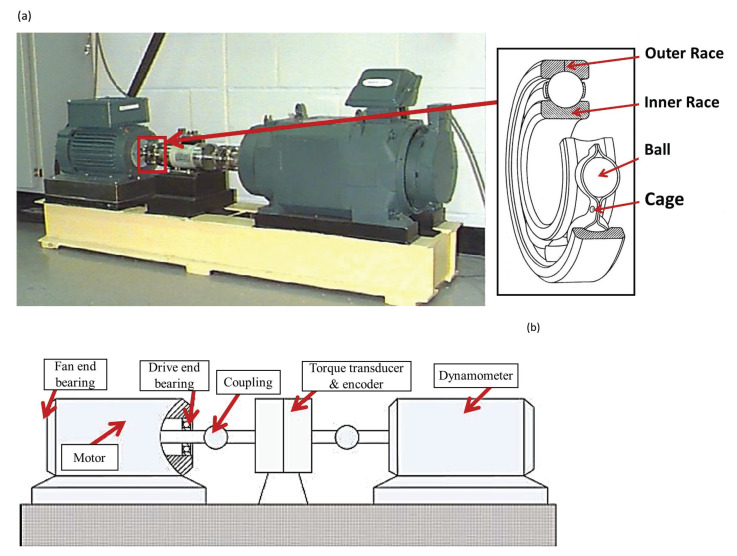
Testbed of the CWRU for bearing defects [42] and the components of REBs: (**a**) Photo of the test bench, (**b**) Structural description of the bench.

The dataset from the CWRU [42] was used to evaluate the proposed methodology. Figure 3 shows the CWRU’s experimental rig for the study of ball bearing defects. Vibration measurements were acquired with three accelerometers placed in the 12 o’clock position on the housing of the drive end (DE) and fan end (FE). SKF deep-groove ball bearings of 6205-2RS JEM and 6203-2RS JEM types were employed for both the DE end FE, respectively. Electro-discharge machining was used to generate different fault diameters ranging from 0.007 to 0.028 inch. Vibration signals were recorded at 12 kHz, under varying motor speeds from 1797 to 1720 rpm and four motor load operating conditions denoted as:

- No-load condition ($L_{0}$): 0% of the nominal load;
- Half-loaded condition ($L_{1}$): 50% of the nominal load;
- Fully loaded condition ($L_{2}$): 100% of the nominal load;
- Overloaded condition ($L_{3}$): 150% of the nominal load;
- Combination of all the load conditions: ($L_{n}$).

The four classes under study were as follows:

- *H*: corresponding to the healthy behaviour (no fault);
- $F_{1}$: faulty case with a severity of 0.007 inch;
- $F_{2}$: faulty case with a severity of 0.014 inch;
- $F_{3}$: faulty case with a severity of 0.021 inch.

The training and testing time were evaluated using a computer operating with Windows 10 pro 64-bit and a processor Intel(R) Core(TM) i7-6500U CPU @ 2.50–2.59 GHz.

### 4.2. Experimental Validation

As depicted in Figure 2, in the first part of this work, the KLD of the retained IMFs were considered as input features for the classification stage. Under a single load condition, 900 realisations of this feature were computed for each feature and for each fault severity level. Taking the example of the unloaded condition $L_{0}$ with one fault severity, the input matrix for the classification was organized as follows:

$$\left\{\begin{array}{cccc} kIMF_{2{,}_{1}} & kIMF_{3{,}_{1}} & kIMF_{4{,}_{1}} & kIMF_{6{,}_{1}}\\ kIMF_{2{,}_{2}} & kIMF_{3{,}_{2}} & kIMF_{4{,}_{2}} & kIMF_{6{,}_{2}}\\ \vdots & \vdots & \vdots & \vdots \\ kIMF_{2{,}_{900}} & kIMF_{3{,}_{900}} & kIMF_{4{,}_{900}} & kIMF_{6{,}_{900}} \end{array}\right\}$$

Its elements can be generalized as $kIMF_{j{,}_{i}}$ to be the *i*th realisation of the KLD for the *j*th IMF.

Therefore, for the fault cases (one healthy and three faulty cases), and the four load conditions, the data matrix size was (14,400 × 4. In the following, two-thirds of the data were used for training, and one-third for testing. Note that every case and condition were equivalently represented either in the training or the testing process.

#### 4.2.1. Linear Classification with PCA

**Figure 4:**
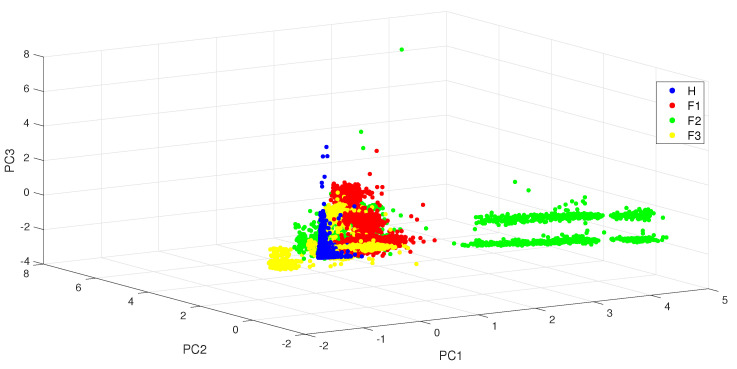
The three-dimensional principal subspace for bearing ball fault data under the different load conditions.

From the PCA contribution rates under different load conditions displayed in Table 3, it can be observed that more than 90% of the information can be captured with the first three principal components. However, the projection of the data in the reduced dimension space shown in Figure 4 reveals a poor classification performance: the different classes have a huge number of overlaps.

In the following sections, nonlinear classifiers are evaluated.

#### 4.2.2. Kernel-Based Classifiers

**Figure 5:**
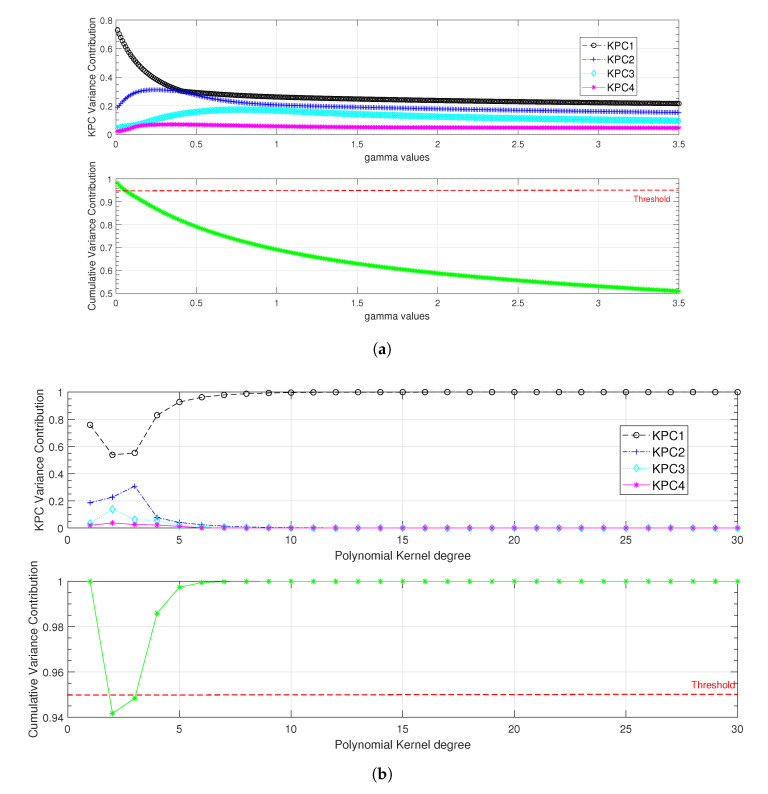
KPCA kernel function hyperparameters adjustment under fully loaded condition. (**a**) Gaussian kernel width parameter regularisation; (**b**) polynomial kernel degree parameter regularisation.

As mentioned in the previous section, the key point for this classifier is the selection and the tuning of the kernel. In the following, Gaussian and polynomial kernels were evaluated. For the Gaussian kernel, we proposed to vary γ from 0.01 to 3.5 with a step of 0.01. For the polynomial kernel, the degree *p* was varied between 1 and 30. The results under fully loaded condition are presented in Figure 5, where a threshold of 95% for the cumulative variance contribution was used to set the most suitable values for each kernel’s parameters.

As shown in Figure 5, the most significant cumulative variance contribution was recorded for the Gaussian kernel with γ equal to 0.01, and for the polynomial kernel with an order p≥6. The degree of the polynomial kernel was set to 6 to minimise the computation time. Once the kernels had been tuned, they were evaluated with all load conditions. The results presented in Table 4 show that with the polynomial kernel, the first kernel principal component (KPC) is of great significance (96.7% of the cumulative variance) while three KPCs are required to reach 91.3% of the feature variation with the Gaussian kernel.

**Figure 6:**
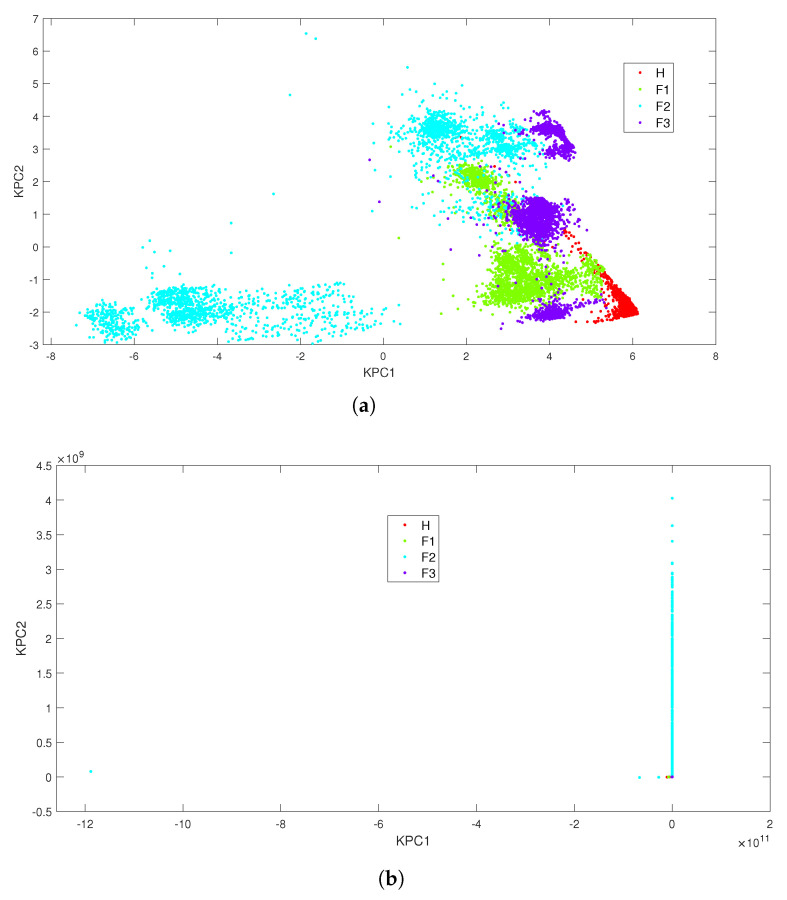
KPCA scatter plot under the all-load-conditions combination. (**a**) Results with Gaussian Kernel (γ=0.01); (**b**) results with polynomial kernel (p=6).

The data projection displayed in Figure 6 shows a poor performance of the classifier whatever the kernel is. A kernel-based technique with a higher dimensional projection space such as a SVM could be an option. However, in previous works [57,64], it was shown that a SVM combined with different approaches (one-against-all, one-against-one and directed acyclic graph SVM) could provide satisfactory classification results. However, these results were obtained to the detriment of high training times ( 132.1 s for the polynomial kernel and 14.05 s for the Gaussian one). Therefore, in the following, systematic data exploration techniques are evaluated: deterministic techniques such as KNN and DT and a probabilistic-based approach (NB).

#### 4.2.3. Classification Results Based on the Systematic Data Exploration Strategy

In this study, deterministic (KNN, DT) and probabilistic (NB) classification techniques were evaluated and compared in terms of the following criteria: training accuracy rate TrA, testing accuracy rate TsA, training time Trt and testing time Tst.

Based on the results reported in Table 5, we can draw several conclusions when using only the KLD of the most sensitive IMFs:

- Under the single-load condition, all the three classifiers exhibited good performance despite a low testing accuracy rate of 96.5% for the NB classifier;
- Under the combined-load condition, the performance of the NB classifier was severely degraded with 82.3% and 81.92% for the training accuracy rate and the testing accuracy rate, respectively.

To improve the classification results, we propose in the following to merge the most relevant features (variance and KLD of $IMF_{2}$ and $IMF_{4}$) as displayed in Table 6.

Three different case studies were considered:

■ Case study with four features
  □ KLD and variance of $IMF_{2}$ and $IMF_{4}$, denoted as C4;
  In this case study, the KLD and the variance of the selected IMFs ($IMF_{2}$ and $IMF_{4}$) were merged together for each load condition as in the following matrix.
  $$\left\{\begin{array}{cccc} kIMF_{2{,}_{1}} & vIMF_{2{,}_{1}} & kIMF_{4{,}_{1}} & vIMF_{4{,}_{1}}\\ kIMF_{2{,}_{2}} & vIMF_{2{,}_{2}} & kIMF_{4{,}_{2}} & vIMF_{4{,}_{2}}\\ \vdots & \vdots & \vdots & \vdots \\ kIMF_{2{,}_{900}} & vIMF_{2{,}_{900}} & kIMF_{4{,}_{900}} & vIMF_{4{,}_{900}} \end{array}\right\}$$
  where $kIMF_{j{,}_{i}}$ and $vIMF_{j{,}_{i}}$ denote the KLD and variance of the *j*th component for their *i*th realisation, respectively.
■ Case study with two features
  □ KLD and variance of $IMF_{2}$, denoted as $C2_{1}$;
  □ KLD and variance of $IMF_{4}$, denoted as $C2_{2}$;
  □ Variances of $IMF_{2}$ and $IMF_{4}$, denoted as $C2_{3}$;
  □ KLD of $IMF_{2}$ and $IMF_{4}$, denoted as $C2_{4}$.
■ Case study with one feature
  □ Variance of $IMF_{2}$, denoted as $C1_{1}$;
  □ Variance of $IMF_{4}$, denoted as $C1_{2}$;
  □ KLD of $IMF_{2}$, denoted as $C1_{3}$;
  □ KLD of $IMF_{4}$, denoted as $C1_{4}$.

Note that for all the case studies, a well balanced two-thirds of the data were used for training, and one-third for testing as mentioned before.

In a first stage, we present the results of the classification when the four features are merged. These results are displayed in Table 7. Compared to those obtained in Table 5, where only the $kIMF_{j{,}_{i}}$ were used, we can notice that the NB’ results are far better. For example, the testing accuracy rate TsA significantly increased from 96.5% to 99.83% under $L_{0}$. The training and testing accuracy rates were almost 100% for KNN and DT under all load conditions. Compared to the previous case study where only the KLD was used, the classification performance was far better when the KLD and variance of the retained IMFs were merged. For the training time and testing time criteria, the variations for all the classifiers were not too significant.

These results are encouraging for bearing ball fault classification using the CWRU Database. In fact, Li et al. in [6], provided a comprehensive benchmark study of the CWRU Database with several entropy-based fault classification methods. They pointed out the difficulty of dealing with bearing ball fault, particularly in the case of F2 (the 0.014-inch fault severity) where most of the classification algorithms exhibited a low performance. In Table 8, we present the comparison results between the multiscale permutation entropy (MPE), highlighted in [6], and our proposed method based on KLD and variance for the specifically selected IMFs.

Our proposed methodology successfully overcame the problem related to the incipient bearing ball fault classification for the CWRU database. The technique successfully reached a 100% average for the testing accuracy rate with KNN and DT classifiers and 99.92% with the NB classifier while the best classification accuracy recorded for the MPE-LR only had an average of 98.75%.

For further analysis, the other combinations of features were analysed to emphasise the overall robustness and sustainability of the proposed procedure. In the following, only the results (training accuracy rate and testing accuracy rate) higher than 99.9% are presented in Table 9. It highlights the best feature, i.e., the most sensitive characteristic to the fault occurrence. For example, in Table 9, it is shown that combining the KLD of $IMF_{2}$ and the variance of $IMF_{4}$ (denoted as C4), the testing accuracy rate is the highest for all the operating points and all the classifiers. The percentages are higher than 99.9%, which shows the effectiveness of the preprocessing and the feature selection.

From these results, several conclusions can be drawn:

- For both training and testing steps, whatever the load condition or the used classifier, case C4 with the combination of KLD and variance for $IMF_{2}$ and $IMF_{4}$ offers the best performance.
- This analysis shows that it is possible to adapt to each case and meet the application requirements. Taking the example of load $L_{3}$, we can choose to work with either four features (C4), two features ($C2_{1}$) or even one feature ($C1_{1}$: variance of $IMF_{2}$) to reach 100% of classification accuracy. This flexibility can address the computation time that is strongly linked to the number of used features corresponding to the input’s dimension of the classification system.
- Finally, we can conclude that in our study, the KNN classifier offers the most efficient combinations of features.

The analysis of the classification accuracy is undoubtedly of great importance to conclude on the efficiency of the preprocessing, extraction and feature analysis steps. However, the evaluation of the computation time for each approach is also an important element for an industrial application. Therefore, after selecting the feature combinations that gave the best test and training accuracy rates, we evaluated the learning and testing times as a function of load condition variation.

**Figure 7:**
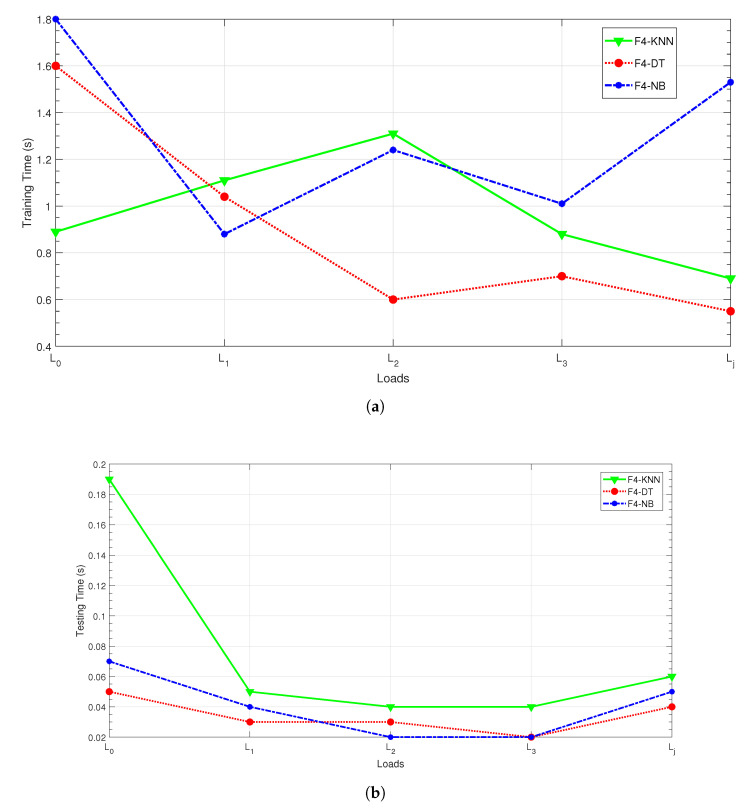
Classification time computation for the C4 feature selection. (**a**) Training time evaluation; (**b**) testing time evaluation.

Only the results with four features (KLD and variance of $IMF_{2}$ and $IMF_{4}$ denoted as C4) are displayed in Figure 7. It can be noticed that for all the classifiers, the computational burden is acceptable with a testing time lower than 200 ms regardless of the load condition. We notice that in the literature most of the techniques only provide the classification accuracy [65,66,67].

## 5. Conclusions

This work presented a methodology for bearing ball fault classification. According to the literature, ball defects are the most difficult to detect because of their position in the bearing. In their early phase, we proposed a specific methodology to proceed to these incipient faults’ efficient detection and classification. Generally, bearing balls can be affected by corrosion, spalling, high temperatures or premature wear due to overloading and this can lead, in the long term, to holes in the balls as considered here. The proposed method was evaluated with the dataset provided by CWRU. The database proposes vibration data under different operating conditions: variable motor rotation speeds (ranging from 1730 rpm to 1797 rpm), several defects with different severity levels (0.007 inch, 0.014 inch and 0.021 inch) and different load conditions ( $L_{0}$, $L_{1}$, $L_{2}$ and $L_{3}$). The proposed methodology successfully classified the incipient ball fault with a classification rate higher than those obtained in previous literature works.

The main idea of this proposed method relied on the preprocessing and feature selection steps. For this purpose, we used an EMD for decomposing the ball vibration signals into finite time–frequency domain bands (IMFs). Then, an energy analysis and KLD evaluation allowed us to retain only the IMFs which were the most fault-sensitive and robust to load variations. Finally, we showed that the variance and the KLD of the retained IMFs were the best features for ball fault classification. As we dealt with real, nonlinear and nonstationary signals, we confirmed that a PCA could not separate the data. Three main nonlinear approaches were investigated including the kernel-based techniques (KPCA or SVM), systematic deterministic (KNN and DT) or probabilistic (NB) data exploration techniques. The performance of the classifiers was evaluated through the following criteria: TrA,TsA,Trt and Tst. The results showed that merging the features led to the highest classification accuracy compared to the data provided in the literature. We also showed that this performance was compatible with industrial applications, regarding the computation time constraints. Due to the lack of industrial manufacturer free-to-use data and methodologies, a deeper comparison with these systems was not possible.

## Figures and Tables

**Table 1:** IMFs’ relative deviation percentage for signal-to-noise ratio.

IMF Rank	SNR RDP (%)
**1**	**5**
**2**	**11.7**
**3**	**21.6**
**4**	**20**
**5**	**17.3**
**6**	**22.3**
**7**	**28.2**
**8**	**34.8**
9	44.9
10	51.8
11	59.2
12	62.1
13	68.2
14	68.9
15	59.1
16	62.1
17	83
18	81.1

**Table 2:** Sensitivity of the different statistical information in multiple operating conditions.

	AUC for Mean				AUC for Variance				AUC for Skewness				AUC for Kurtosis				AUC for KLD			
IMF	$L_{0}$	$L_{1}$	$L_{2}$	$L_{3}$	$L_{0}$	$L_{1}$	$L_{2}$	$L_{3}$	$L_{0}$	$L_{1}$	$L_{2}$	$L_{3}$	$L_{0}$	$L_{1}$	$L_{2}$	$L_{3}$	$L_{0}$	$L_{1}$	$L_{2}$	$L_{3}$
2	0.971	0.982	1	0.998	1	0.996	1	1	0.7	0.838	0.835	0.65	1	0.955	0.996	0.878	1	1	1	1
3	0.853	0.968	0.602	0.836	1	0.998	1	1	0.816	0.996	0.911	0.962	0.824	0.845	0.784	0.638	1	1	1	1
4	0.937	0.82	0.801	0.84	1	0.968	1	1	0.882	0.733	0.872	0.602	1	0.975	1	0.993	1	1	1	1
5	0.586	0.773	0.999	0.729	1	0.967	1	1	0.587	0.554	0.674	0.963	0.926	0.966	1	1	1	1	1	1
6	0.889	0.71	0.979	0.953	1	0.995	1	1	0.776	0.681	0.634	0.705	1	0.769	0.917	0.913	1	1	1	1
7	0.649	0.634	0.783	0.958	1	0.962	1	1	0.565	0.51	0.632	0.6	0.968	0.862	0.994	0.733	0.666	0.457	0.842	0.911
8	0.517	0.623	0.637	0.651	1	0.972	1	1	0.552	0.687	0.674	0.622	0.534	0.733	0.917	0.976	0.49	0.45	0.901	0.5

*L*_0_: no-load condition, *L*_1_: half-load condition, *L*_2_: full-load condition, *L*_3_: overload condition.

**Table 3:** PCA contribution rates evaluation under different load conditions.

Load Condition	PC	Eigenvalue	Variance Contribution (%)	Cumulative Variance (%)
$L_{0}$	**1**	2.638	65.956	**65.956**
	**2**	0.982	24.572	**90.53**
	**3**	0.277	6.932	**97.46**
	4	0.105	2.537	100
$L_{1}$	**1**	1.672	41.814	**41.814**
	**2**	1.475	36.847	**78.69**
	**3**	0.482	12.071	**90.76**
	4	0.369	9.239	100
$L_{2}$	**1**	3.073	75.927	**75.927**
	**2**	0.744	18.617	**94.55**
	**3**	0.136	3.422	**97.97**
	4	0.081	2.031	100
$L_{3}$	**1**	2.706	67.653	**67.653**
	**2**	1.039	25.996	**93.65**
	**3**	0.172	4.31	**97.96**
	4	0.081	2.01	100
$L_{n}$	**1**	1.829	45.747	**45.747**
	**2**	1.132	28.322	**74.07**
	**3**	0.806	20.151	**94.22**
	4	0.231	5.779	100

The bold values highlights the main PC used in the study with Figure 4.

**Table 4:** KPCA contribution rates evaluation with Gaussian and polynomial kernels.

KPC	Variance Contribution		Cumulative Variance (%)	
	Gaussian Kernel	Polynomial Kernel	Gaussian Kernel	Polynomial Kernel
1	0.433	0.967	43.3	96.7
2	0.281	0.23	71.4	99
3	0.199	0.007	91.3	99.7
4	0.087	0.003	100	100

The bold values highlights the main PC used in the study with Figure 6.

**Table 5:** Comparison of bearing ball fault classification using KLD of the IMFs.

Classifier	KNN					DT					NB				
Load (hp)	$L_{0}$	$L_{1}$	$L_{2}$	$L_{3}$	$L_{n}$	$L_{0}$	$L_{1}$	$L_{2}$	$L_{3}$	$L_{n}$	$L_{0}$	$L_{1}$	$L_{2}$	$L_{3}$	$L_{n}$
Training accuracy rate (%)	98.2	99.8	99.1	100	99	98.3	100	98.6	100	98.6	98	99.3	98	99.6	**82.3**
Testing accuracy rate (%)	97.42	99.92	98.91	99.75	98.83	98.25	99.92	98.33	99.75	98.15	96.5	99.42	97.83	99.83	**81.92**
Training time (s)	0.31	0.9	0.31	0.3	0.56	0.28	0.24	0.25	0.25	1.15	0.65	0.75	0.7	0.67	1
Testing time (s)	0.03	0.02	0.03	0.02	0.15	0.02	0.02	0.01	0.02	0.05	0.05	0.02	0.02	0.02	0.05

The colors highlight the results for the three Machine Learning techniques.

**Table 6:** Final feature selection.

Features	Relevant IMFs			
Variance	$IMF_{2}$	$IMF_{3}$	$IMF_{4}$	
KLD	$IMF_{2}$	$IMF_{3}$	$IMF_{4}$	$IMF_{6}$

**Table 7:** Classification results using KLD and variance of $IMF_{2}$ and $IMF_{4}$.

Classifier	KNN					DT					NB				
Load (hp)	$L_{0}$	$L_{1}$	$L_{2}$	$L_{3}$	$L_{n}$	$L_{0}$	$L_{1}$	$L_{2}$	$L_{3}$	$L_{n}$	$L_{0}$	$L_{1}$	$L_{2}$	$L_{3}$	$L_{n}$
Training accuracy rate (%)	100	100	99.9	100	100	100	100	99.9	100	100	99.9	100	99.9	100	100
Testing accuracy rate (%)	100	100	100	100	100	100	100	100	100	100	99.83	100	99.85	100	100
Training time (s)	0.89	1.1	1.31	0.88	0.69	1.58	1.04	0.55	0.56	0.55	1.8	0.87	1.24	1.01	1.53
Testing time (s)	0.19	0.05	0.03	0.04	0.06	0.05	0.03	0.03	0.02	0.04	0.07	0.04	0.02	0.02	0.05

The colors highlight the results for the three Machine Learning techniques.

**Table 8:** Classification comparative results.

Ref	Fault Type		Ball					
	Load (hp)		$L_{0}$	$L_{1}$	$L_{2}$	$L_{3}$	$L_{n}$	Mean
	Algorithm		Testing Accuracy Rates (%)					
[6]	MPE	KNN	93	99	100	100	Not provided	98
		SVM	81	99	100	98	Not provided	94.5
		Logic regression	96	99	100	100	Not provided	98.75
		Backpropagation NN	70	91	90	93	Not provided	86
		Extreme learning Machine	92	90	100	100	Not provided	97.5
		Soft regression	94	99	100	100	Not provided	98.25
Proposed technique	KLD and variance	KNN	100	100	100	100	100	100
		DT	100	100	100	100	100	100
		NB	99.83	100	99.85	100	100	99.92

**Table 9:** Best feature combinations according to the classification accuracy under different load conditions.

	KNN		DT		NB	
Load Condition	TrA(%)	TsA(%)	TrA(%)	TsA(%)	TrA(%)	TsA(%)
$L_{0}$	C4	C4	C4	C4	C4	C4
	$C2_{1}$	$C2_{1}$	$C2_{1}$	$C2_{1}$	$C2_{1}$	$C2_{1}$
			$C2_{3}$		$C2_{3}$	
$L_{1}$	C4	C4	C4	C4	C4	C4
	$C2_{3}$	$C2_{2}$	$C2_{2}$	$C2_{2}$	$C2_{2}$	$C2_{2}$
	$C1_{2}$	$C2_{3}$	$C2_{3}$	$C2_{3}$	$C2_{3}$	$C2_{3}$
		$C2_{4}$		$C1_{2}$		$C1_{2}$
		$C1_{2}$				
$L_{2}$	C4	C4	C4	C4	C4	C4
	$C2_{3}$	$C2_{2}$	$C2_{3}$	$C2_{3}$	$C2_{3}$	$C2_{3}$
	$C1_{2}$	$C2_{3}$	$C1_{2}$	$C1_{2}$	$C1_{2}$	
		$C1_{1}$				
		$C1_{2}$				
$L_{3}$	C4	C4	C4	C4	C4	C4
	$C2_{1}$	$C2_{1}$	$C2_{1}$	$C2_{1}$	$C2_{1}$	$C2_{1}$
	$C2_{3}$	$C2_{3}$	$C2_{3}$	$C2_{3}$	$C2_{3}$	$C2_{3}$
	$C1_{1}$	$C2_{4}$	$C1_{1}$	$C1_{1}$	$C1_{1}$	$C1_{1}$
		$C1_{1}$				
$L_{n}$	C4	C4	C4	C4	C4	C4
	$C2_{1}$	$C2_{1}$				
	$C2_{3}$	$C2_{2}$				
		$C2_{3}$				
		$C2_{4}$				
		$C1_{1}$				
		$C1_{2}$				
		$C1_{4}$				

The color highlights the best feature combination for each load condition.
